# Discovering disease–disease associations using electronic health records in The Guideline Advantage (TGA) dataset

**DOI:** 10.1038/s41598-021-00345-z

**Published:** 2021-10-25

**Authors:** Aixia Guo, Yosef M. Khan, James R. Langabeer, Randi E. Foraker

**Affiliations:** 1 Institute for Informatics (I2), Washington University School of Medicine, St. Louis, MO USA; 2 Health Informatics and Analytics, Centers for Health Metrics and Evaluation, American Heart Association, Dallas, TX USA; 3 School of Biomedical Informatics, Health Science Center at Houston, The University of Texas, Houston, TX USA; 4 Department of Internal Medicine, Washington University School of Medicine, St. Louis, MO USA

**Keywords:** Diseases, Health care

## Abstract

Certain diseases have strong comorbidity and co-occurrence with others. Understanding disease–disease associations can potentially increase awareness among healthcare providers of co-occurring conditions and facilitate earlier diagnosis, prevention and treatment of patients. In this study, we utilized the valuable and large The Guideline Advantage (TGA) longitudinal electronic health record dataset from 70 outpatient clinics across the United States to investigate potential disease–disease associations. Specifically, the most prevalent 50 disease diagnoses were manually identified from 165,732 unique patients. To investigate the co-occurrence or dependency associations among the 50 diseases, the categorical disease terms were first mapped into numerical vectors based on disease co-occurrence frequency in individual patients using the Word2Vec approach. Then the novel and interesting disease association clusters were identified using correlation and clustering analyses in the numerical space. Moreover, the distribution of time delay (*Δt*) between pair-wise strongly associated diseases (correlation coefficients ≥ 0.5) were calculated to show the dependency among the diseases. The results can indicate the risk of disease comorbidity and complications, and facilitate disease prevention and optimal treatment decision-making.

## Introduction

Many diseases have strong associations with others and often co-occur within patients. Comorbidities are particularly common among very sick and older patients. Healthcare providers may not be fully aware of the associations between different diseases. Ignoring relationships between diseases may preclude healthcare providers from providing timely diagnoses and effective treatments and prevention^1^. Along with the increasing generation and availability of large-scale genomics data, and digitized electronic health records (EHR) data, a few studies have been reported to investigate disease–disease associations and relationships^2–7^. For example, the Disease Ontology (DO)^8^ is a widely used database to annotate the disease associations. The DO annotations and relationships are built based on the disease information collected from multiple data resources, e.g., Medical Subject Headings (MeSH)^9^, Online Mendelian Inheritance in Man (OMIM)^10^, International Classification of Diseases (ICD)^11^, National Cancer Institute (NCI’s) thesaurus^12^, and Systematized Nomenclature of Medicine Clinical Terms (SNOMED CT)^13^. In addition, the large-scale and diverse genomics and molecular datasets were systematically integrated and analyzed to uncover disease–disease associations^14^. For example, 14 novel disease–disease associations were identified by integrating systems-level molecular data^14^. Moreover, EHR datasets contain rich longitudinal healthcare information such as diagnoses, medications and are therefore suitable to discover new disease–disease associations^15^. For example, large-scale EHR datasets (including 35 million patients) were analyzed and combined with the genome-wide association study (GWAS) data (indicating the disease–gene associations) to uncover novel disease–disease and disease–gene associations^7^.

The contribution of this study is to uncover novel disease associations utilizing a large-scale, and nationally representative long-term and longitudinal EHR dataset, i.e., The Guideline Advantage (TGA) dataset. TGA is a clinical data registry that was established in 2011 and was jointly operated by the American Cancer Society, the American Diabetes Association, and the American Heart Association, which covers diverse disease conditions and thus is a suitable data resource for studying disease–disease associations^16^. The program collected longitudinal EHR data from 70 ambulatory clinics across the United States to track and monitor outpatient prevention and disease management among 362,533 unique patients in TGA data set. Different from in-hospital patients’ EHR data, the TGA data provides a unique resource to understand the potential long-term, like hundreds of days, disease–disease associations, which are important for disease diagnosis, prevention, and treatment decision making.

In this exploratory study, we identified the most prevalent 50 disease diagnoses from 165,732 unique patients. Then a set of novel and interesting co-occurrence and dependency associations among these diseases were identified by mapping the categorical disease terms into a numerical space using the Word2Vec^17^ approach followed by the correlation and clustering analyses. Moreover, we investigated the distribution of time delay (*Δt*) of co-occurrence or dependency between 32 strongly associated disease pairs, to understand the risk of comorbidity and complications, and facilitate disease prevention and optimal treatment decision-making.

## Method

### Data source and study population design

The Guideline Advantage (TGA) is a clinical data registry which was established in 2011 and was jointly operated by the American Cancer Society, the American Diabetes Association, and the American Heart Association^16^. The TGA collected longitudinal EHR data from over 70 ambulatory clinics across the United States to track and monitor outpatient prevention and disease management among 362,533 unique patients in TGA data set.

There are 19,599 unique ICD-9 and ICD-10 codes in TGA data. We first converted all the ICD-9 and ICD-10 codes to a smaller number of clinically meaningful categories of diagnoses using Clinical Classifications Software (CCS)^18^. After the codes were converted to the appropriate CCS category, 284 unique single-level CCS category codes have remained. Among all these CCS codes, we selected all patients who had at least one of the most prevalent 50 codes (n = 285,408). In order to study the associations between different diseases within patients, we selected from this subset of patients those with more than five unique CCS category codes (n = 165,732). Patients were tracked for a median of 3 years with a standard deviation of 0.5 years on all locations. Table S1 showed the CCS disease group statistics for all the studied 50 CCS codes in “Supplementary Material”.

### Data analysis

For individual patients, their CCS category codes were ordered in the chronological order. Then the 50 codes were mapped to a 32-dimensional vector space using Word2Vec^17^, i.e., each CCS code was represented by a 32-dimensional numerical vector. Specifically, we used a continuous bag of words model to predict a target word from a window of neighboring words. The Python Gensim Word2Vec model was used with the following hyperparameters: size (embedding dimension) was 32, window (the maximum distance between a target word and all words around it) was 5, min_count (the minimum number of words counted when training the model) was 1, sg (the training algorithm) was CBOW (The continuous bag of words). Different hyperparameters (window, min_counts, embedding dimension) were tested. We then calculated the correlation coefficient matrix for different values of hyperparameters, and then we compared them and selected the hyperparameters based on the similarity shown by heatmap and line plots (see Figure S1).

**Figure 1:**
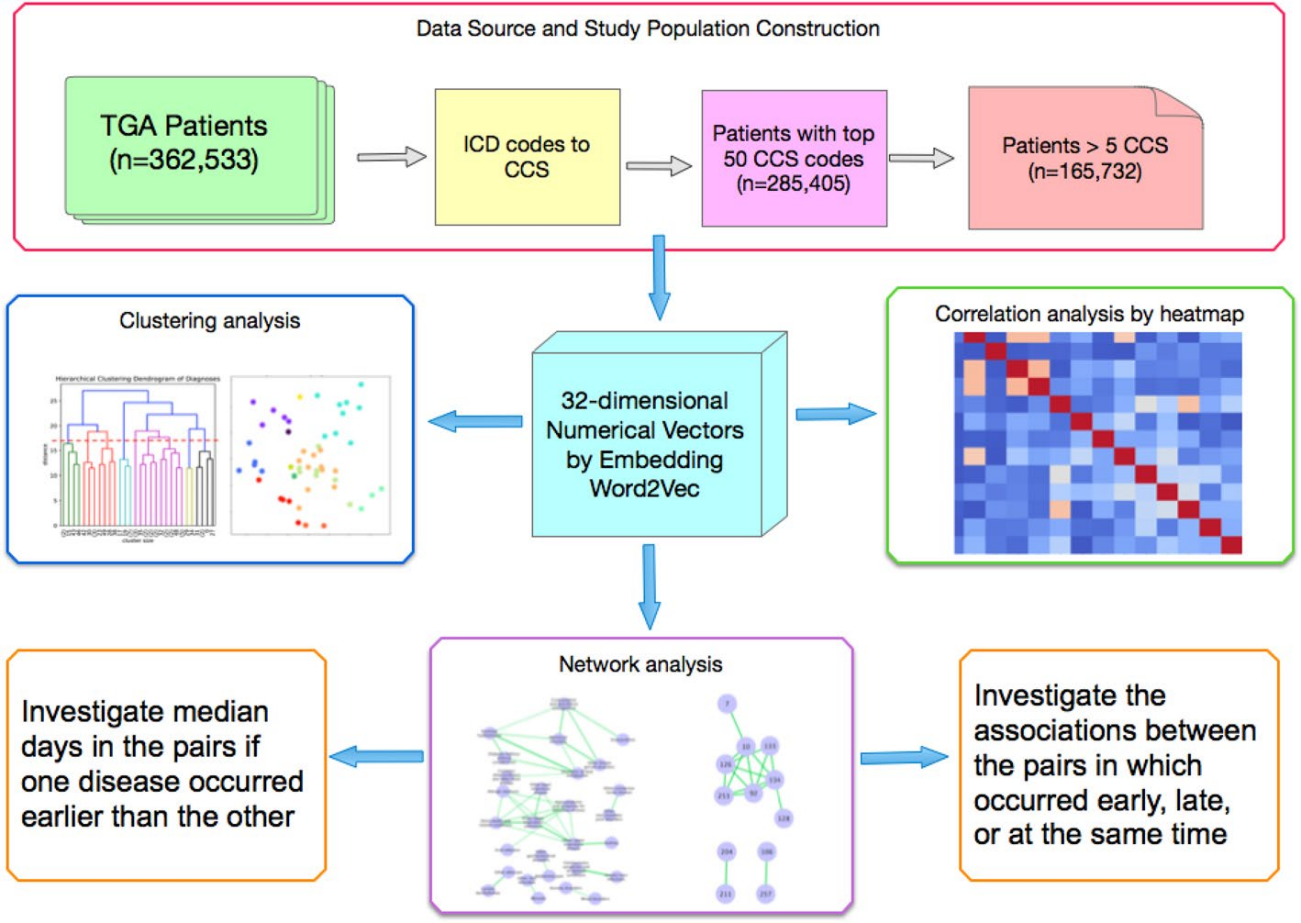
Overview flowchart of the proposed analyses.

After we obtained these numerical vectors of the 50 CCS codes, the spatial distribution of these codes was displayed in a two-dimensional (2D) space using principal component analysis (PCA)^19^. We also investigated the correlations between different CCS codes by heatmap^20^ and conducted a hierarchical clustering analysis^21^ to investigate the similarities between different diseases. Both the correlation and hierarchical clustering analysis were done by using the original embedding numerical vectors. Finally, we conducted network analysis^22^ for the diseases that had strong correlations and compared the results from DO. DO was developed as a standardized ontology for human diseases by the University of Maryland School of Medicine, Institute for Genome Sciences. It provides the descriptions of human disease terms, phenotype characteristics and disease–disease associations. From the website of disease ontology (https://disease-ontology.org/), the disease–disease associations can be retrieved by using the “Search” and “Visualize” functions of a given disease ontology. We also investigated which of the disease pair usually occurred earlier and also the median number of days separating the diagnoses. Figure 1 shows the overview flowchart of the proposed analyses.

## Results

### Characteristics of the study population

The TGA population was 57% female and 48% white (see Table 1). The average age was around 50 years with a standard deviation of 22 years. “Essential hypertension” (43%), “Disorders of lipid metabolism” (40%), and “Other nutritional; endocrine; and metabolic disorders” (33%) are among the top ten CCS diagnosis codes, based on the occurrence frequency (see Table 1). Also, there are many patients have respiratory infections/diseases in the TGA population.

**Table 1:** Characteristics [mean (SD) or n (%)] of the study population.

Demographics	
Total patients *n*	165,732
Age *n (std)*	50 (22)

	*n (%)*
**Gender**	
Female	95,047 (57.3)
Male	70,625 (42.6)
Other/unknown	60 (0.0)
**Race**	
White	78,990 (47.7)
Non-White	31,037 (18.7)
Unknown	56,295 (34.0)
**Top 10 most prevalent CCS categories**	
Essential hypertension	71,895 (43.4)
Disorders of lipid metabolism	65,622 (39.6)
Immunizations and screening for infectious disease	59,050 (35.6)
Other nutritional; endocrine; and metabolic disorders	55,365 (33.4)
Other screening for suspected conditions (not mental disorders or infectious disease)	52,225 (31.5)
Other upper respiratory infections	47,656 (28.8)
Other upper respiratory disease	38,246 (23.1)
Other lower respiratory disease	37,037 (22.3)
Spondylosis; intervertebral disc disorders; other back problems	36,374 (21.9)
Screening and history of mental health and substance abuse codes	36,373 (21.9)

### Disease/diagnosis comorbidity analysis

**Figure 2:**
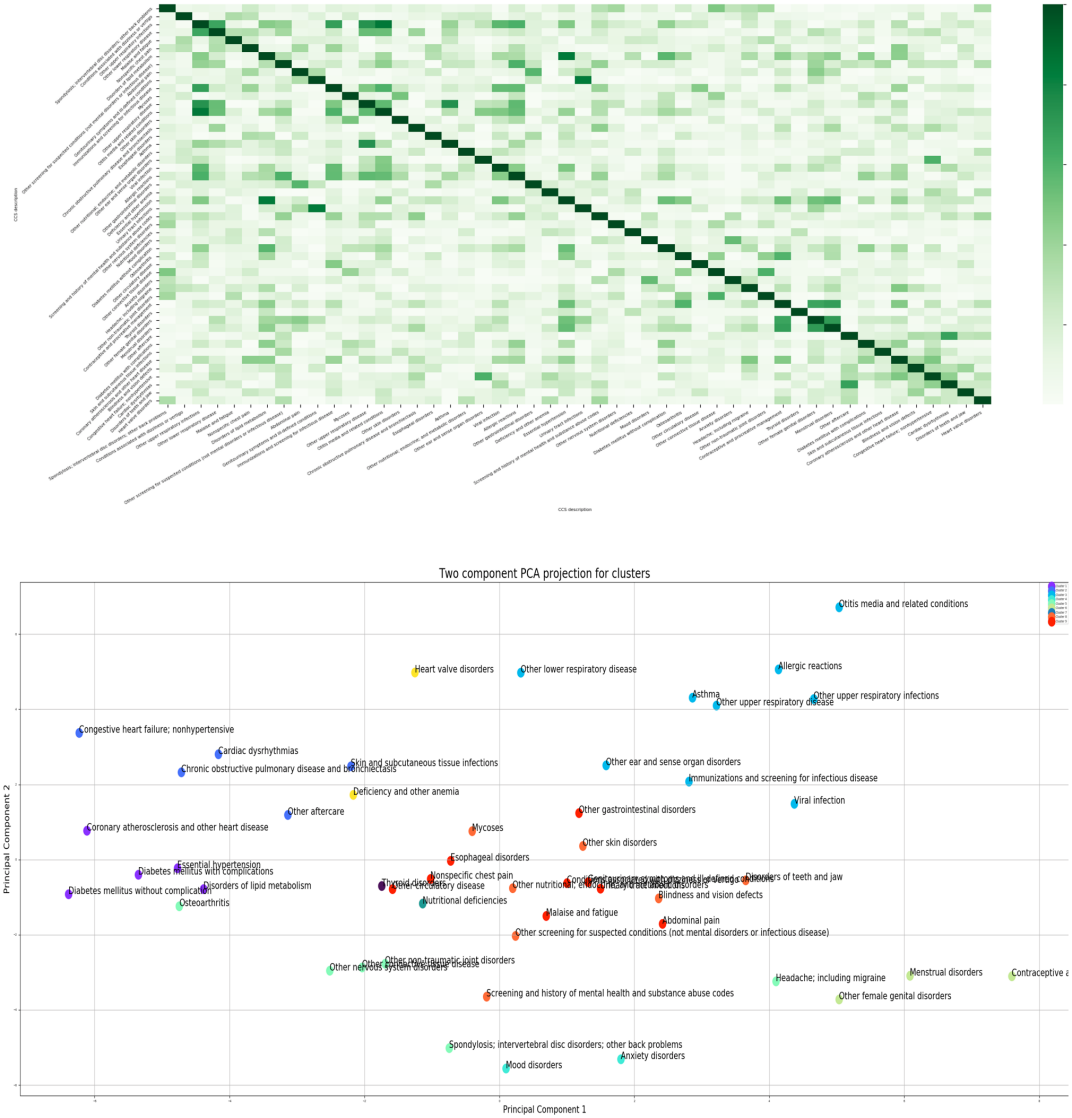
Correlation and clustering analyses of the 50 CCS diagnosis codes. The figures were generated by using Python 3.6.8. with package of Matplotlib.

The word2vec analysis convert the CCS disease/diagnosis codes into numerical vectors based on the CCS code co-occurrence frequency. Thus, the Pearson correlation of the numerical vectors can indicate their comorbidity among the 50 diseases. Figure 2-upper panel shows the correlation between the 50 CCS codes on a heatmap, with darker color indicating a stronger correlation. For example, Genitourinary symptoms and ill-defined conditions and Urinary tract infections showed a strong correlation (correlation coefficient = 0.83).

To better visualize and understand the disease/diagnosis co-occurrence in the TGA population, we conducted the clustering analysis. Figure 2-lower panel shows the disease clustering analysis results and visualization on the 2D PCA projection of the 32-dimensional vectors. Smaller distances indicate closer relationships. Using hierarchical clustering analysis^21^, nine clusters were identified for the 50 CCS codes. For example, the three diseases at the right bottom: contraceptive and procreative management, Menstrual disorders, and other female genital disorders are in the same group.

**Figure 3:**
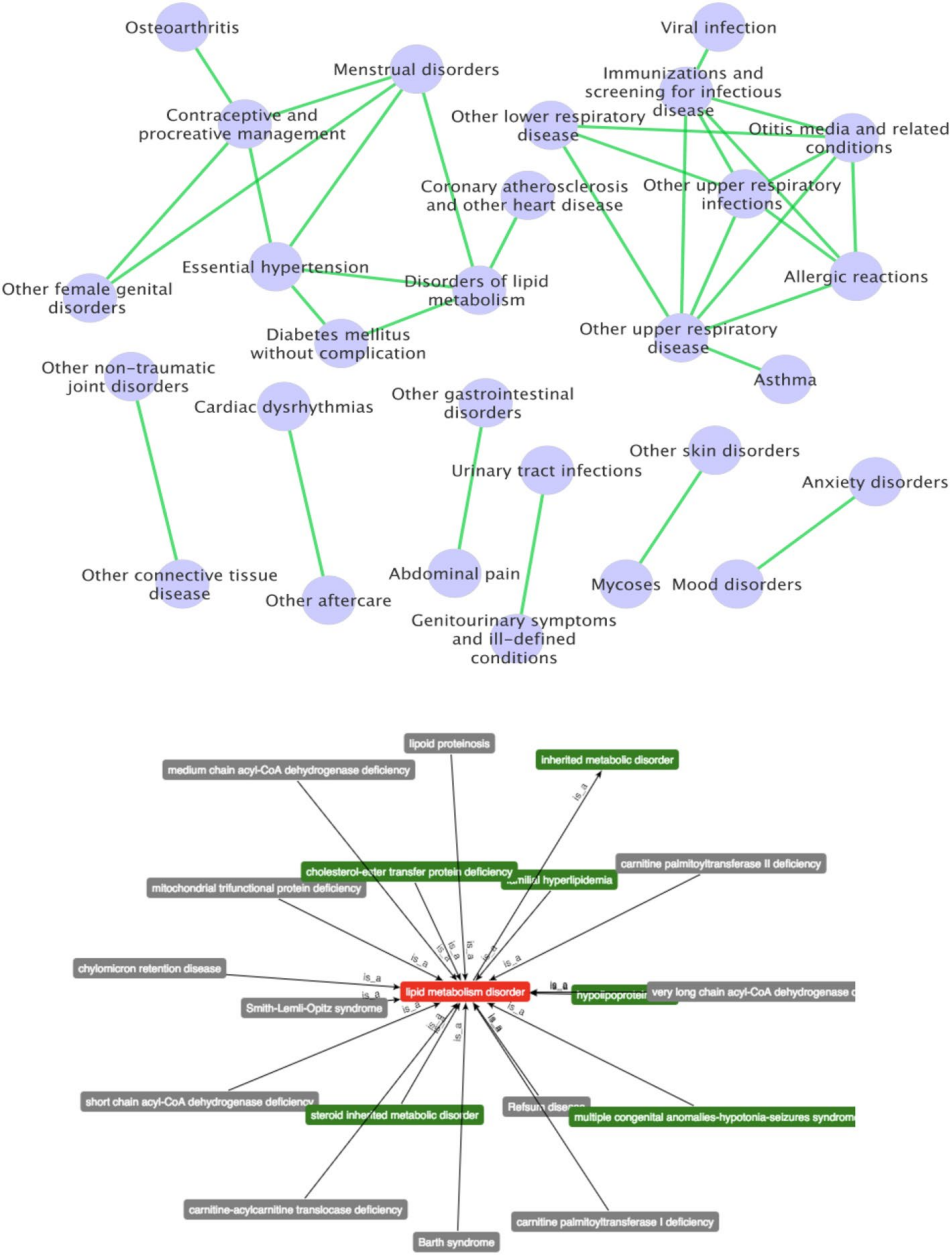
Network analysis of different diseases with strong correlations (top) and the network generated from Disease Ontology (DO) database for an example of disease—‘Disorders of lipid metabolism’ (bottom). The top figure was generated by using Cytoscape (version 3.7.1) software (https://cytoscape.org/). The bottom figure was generated by using public DO database (https://disease-ontology.org).

We further investigated the strong correlation CCS code pairs with the Pearson correlation coefficient ≥ 0.5, and represent the CCS code pairs using network format, as shown in Fig. 3-upper panel. There are 32 edges (CCS code pairs) were remained. As can be seen, some diseases had strong correlations with multiple disease/diagnosis codes. For example, Disorders of lipid metabolism had a strong association with Diabetes mellitus without complication, Essential hypertension, Menstrual disorders, and Coronary atherosclerosis and other heart disease. Whereas, the ‘upper respiratory diseases’ and ‘lower respiratory disease’ and related ‘infections’ often co-occurred. These connections can be helpful for the disease prevention and treatment.

We investigated the network connections of ‘disorders of lipid metabolism’ using the disease ontology (DO) database (see Fig. 3-lower panel). The results indicated that there are 18 different diseases such as inherited metabolic disorder, barth syndrome, and chylomicron retention disease were associated with ‘Disorders of lipid metabolism’. However, it does not include the connections uncovered using the TGA data, which indicate that the TGA data provide unique and new knowledge of disease comorbidity information compared with existing general disease associations.

### Distribution of time delay (Δt) analysis of pair-wise co-occurrence diseases

**Figure 4:**
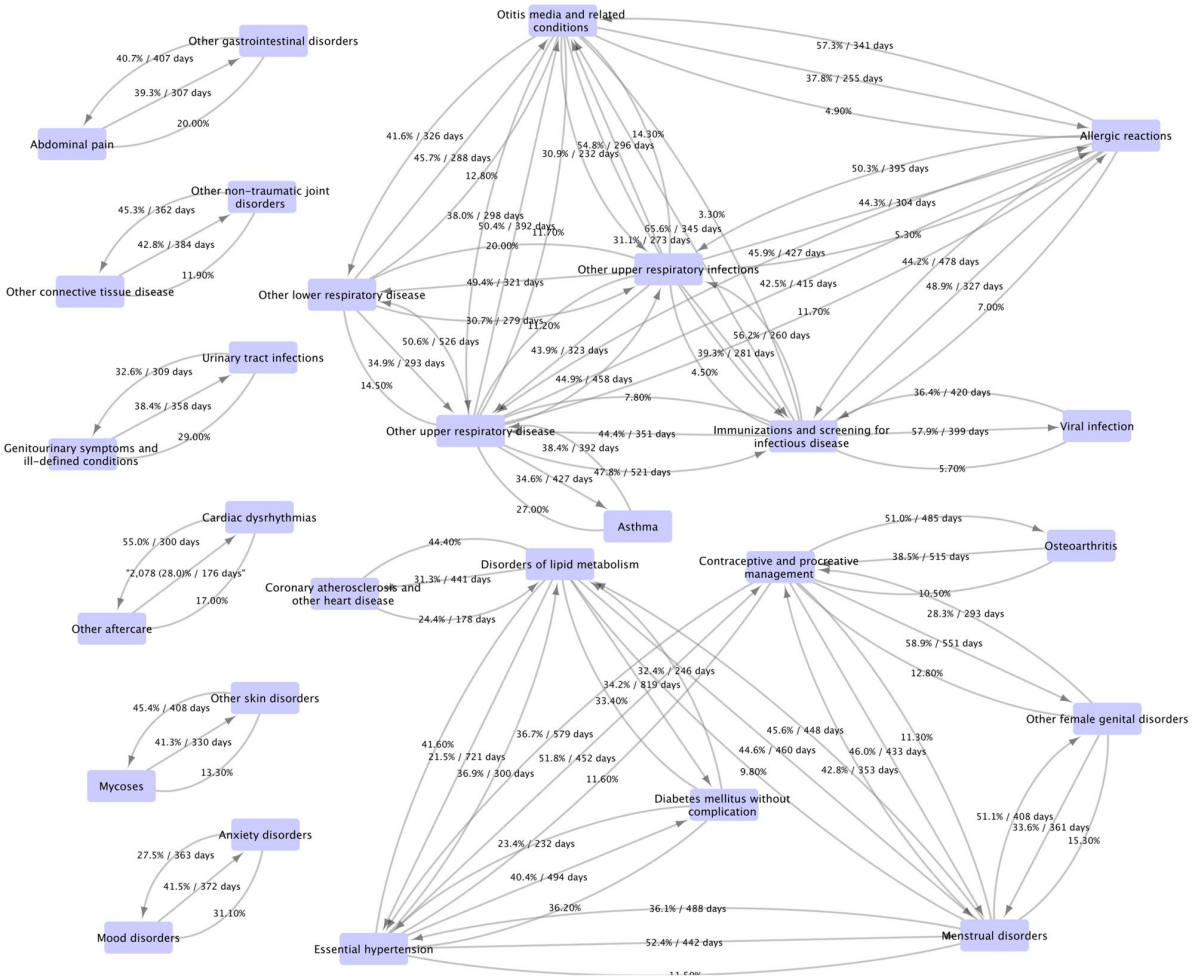
Characteristics of disease pairs using network graphs with strong correlations: percentages and median days of early occurrence. The arrow direction indicates the disease occurrence time order. No arrow means two diseases diagnosed at the same date. The figure was generated by using Cytoscape (version 3.7.1) software (https://cytoscape.org/).

To better understand and answer the question of “when the second disease/diagnosis code happen, once the first disease/diagnosis code happened”, we conducted the distribution of time delay analysis of the 32 strong disease/diagnosis associations. Figure 4 shows the percentages and median days difference between pairs of disease and diagnosis CCS codes, which occurred earlier, later, or at the same time (diagnosed in the same day). For example, among the pair of ‘Abdominal pain’ and ‘Other gastrointestinal disorders’, approximately 39% (41%) patients had ‘Abdominal pain’ earlier (later) than ‘Other gastrointestinal disorders’ and the median days’ difference from ‘Abdominal pain’ earlier (later) to ‘Other gastrointestinal disorders’ was 307 (407) days. And the remaining 20% patients had a diagnosis of ‘Abdominal pain’ and ‘Other gastrointestinal disorders’ on the same date. As we can see, the TGA population data provide the unique view of ‘long-term’ (like hundreds of days) co-occurrence between the 50 disease/diagnosis codes, which means that the prevention treatments can be helpful.

**Figure 5:**
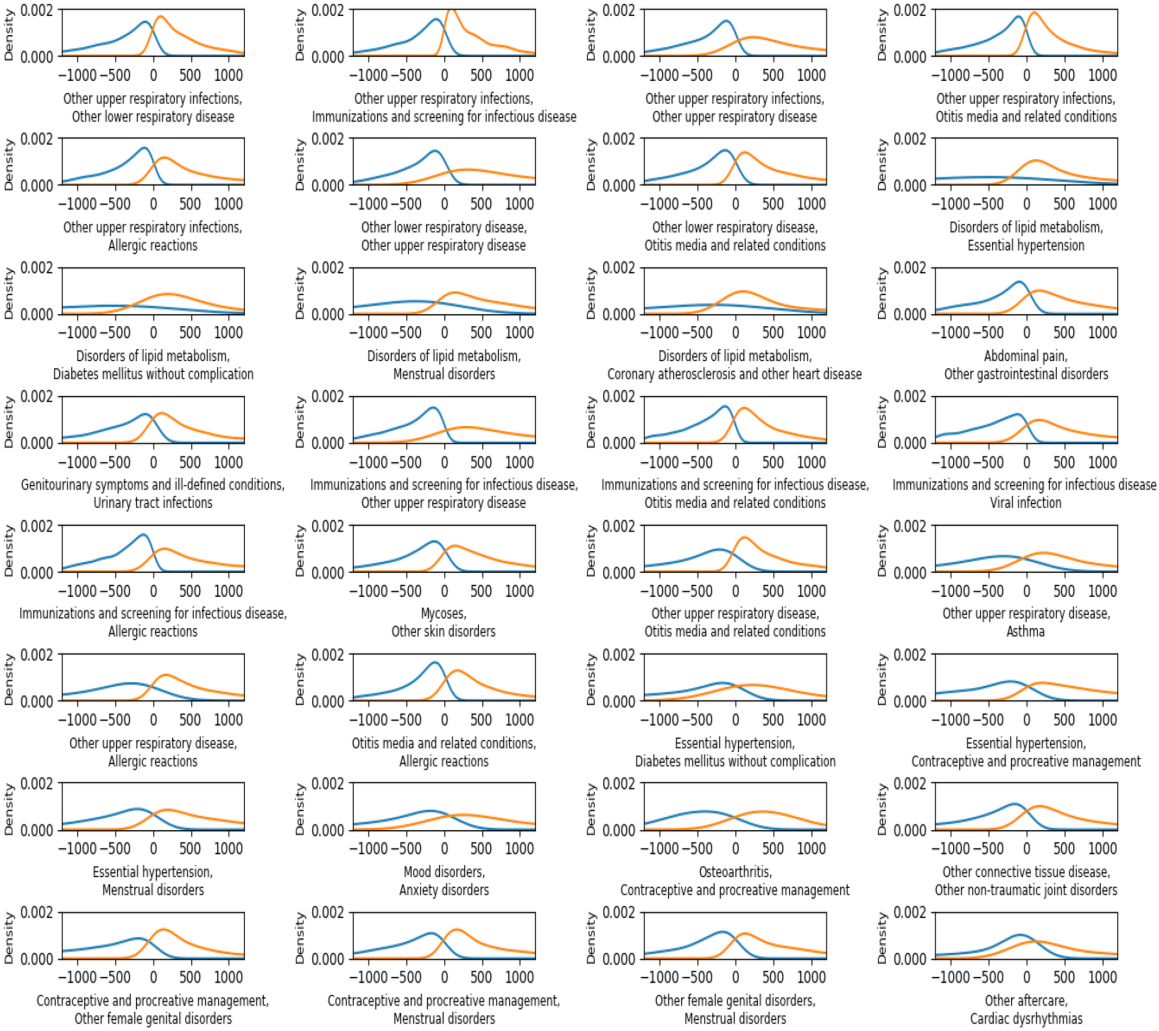
distribution of time delay (*Δt*) for the co-occurrence disease/diagnosis pairs in Fig. 4. The blue curve is about the case of disease 1 occurred earlier than disease 2 and the orange curve is about the case of disease 2 is earlier than disease 1. The figure was generated by using Python 3.6.8 with package of Matplotlib.

Since the median or mean difference can only provide a partial information (of the random variable *Δt*) of the time-delay of the disease/diagnosis co-occurrence, we investigated the distribution of the time-delay *Δt*, using kernel density estimation (KDE)^23^ of the occurrence difference in days for all patients. The analysis results are shown in Fig. 5. The blue curve was about distribution of time delay (*Δt*) for the case of disease 1 occurred earlier than disease 2, while the orange curve was for the case of disease 2 earlier than disease 1. The distribution figures can provide a better understanding of the time-delay. For example, some pairs of disease/diagnosis codes co-occurred in a shorter time and symmetrically (see the “sharp distribution peaks” of the red and blue curves in Fig. 5). Whereas, some pairs of disease/diagnosis codes co-occurred uniformly in a long time period (see the “flatten distribution peaks” of the red and blue curves in Fig. 5).

**Figure 6:**
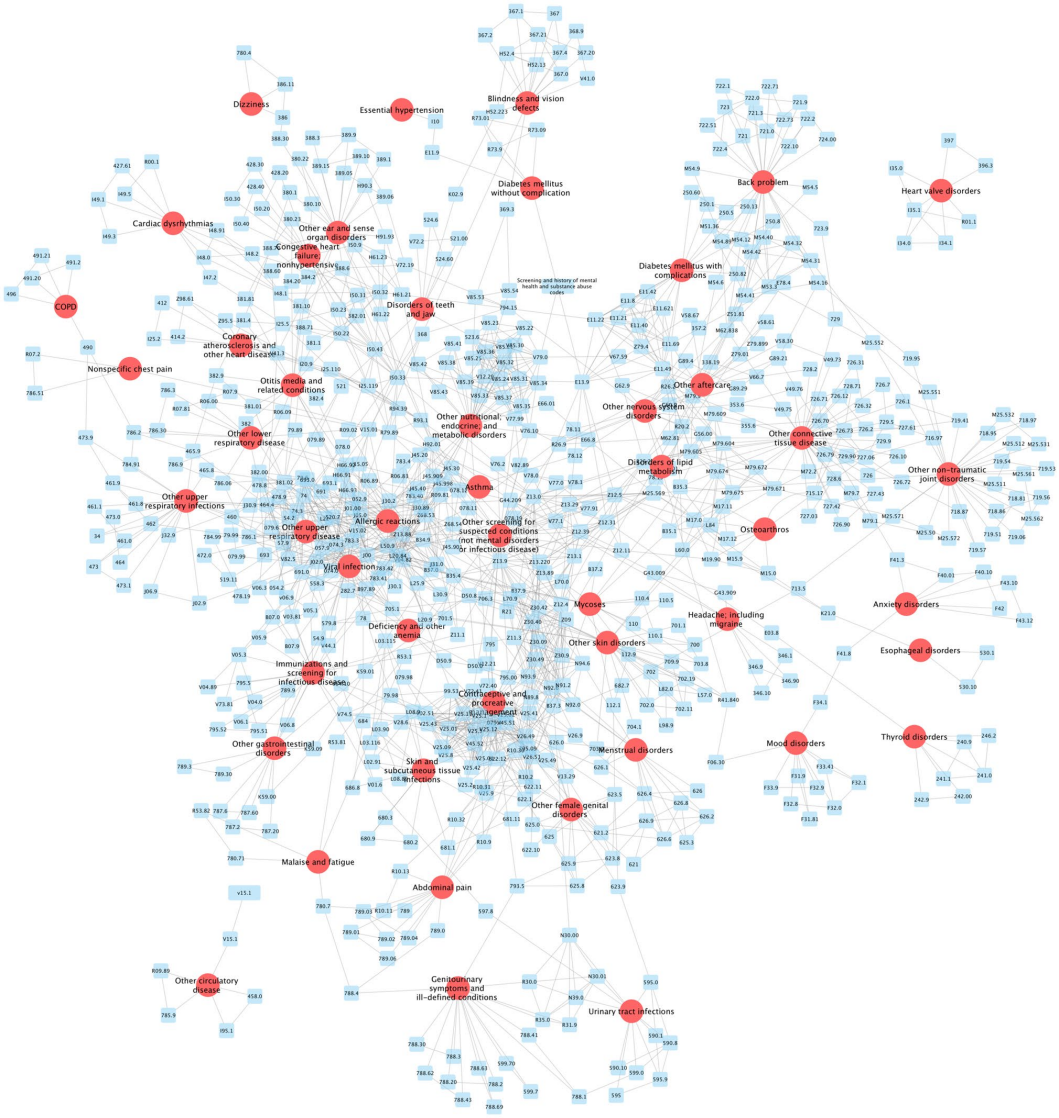
Characteristics of strong ICD codes pairs (Pearson correlation coefficient ≥ 0.8) and disease associations using network graphs. The figure was generated by using Cytoscape (version 3.7.1) software (https://cytoscape.org/).

To investigate the finer disease associations at the ICD code level, the association analysis was conducted on medically-relevant ICD-9 and ICD-10 codes (see Fig. 6). Specifically, the ICD code interactions which had a Pearson correlation coefficient ≥ 0.8 were selected. There were 1412 ICD code interactions among 656 ICD codes. As seen, the ICD code interactions confirmed the aforementioned disease associations, and provided more disease associations. In addition, compared with the disease association results, the ICD code associations with the disease information, as shown in Fig. 6, provided additional and finer information for the disease associations. For example, mood disorders and anxiety disorders were connected via the F34.1 (dysthymic disorder) and F41.8 (anxiety depression (mild or not persistent)) ICD codes. The essential hypertension and diabetes mellitus without complication were associated via the ICD code I10 (essential (primary) hypertension) and E11.9 (type 2 diabetes mellitus without complications). The chronic obstructive pulmonary disease (COPD) and the other upper respiratory infections were connected via code 490 (bronchitis, not specified as acute or chronic) and code 473.9 (unspecified sinusitis (chronic)). There are many more such indications of disease associations via the complex ICD interactions, which can be useful to future study disease associations.

## Discussion

In this study, we utilized longitudinal TGA EHR data from 70 clinics across US to discover associations between 50 different disease categories by word embedding techniques. We displayed by heatmap the correlations between different diseases with numerical vectors obtained from word embeddings. We also conducted a clustering analysis to discover which diseases were in the same co-occurrence cluster. Finally, we analyzed disease pairs with strong correlations by network analysis to show which disease usually occurred earlier and how many days earlier.

Our results indicated that strong associations existed between different diseases. For example, strong associations were discovered between Disorders of lipid metabolism, Diabetes mellitus without complication, and Essential hypertension. From the particular disease example of Disorders of lipid metabolism, we found some new associations and they differed the 18 associated diseases obtained from DO database. To some extent, it showed that the nationally represented TGA data might provide more new information about the disease–disease associations. There were no associated diseases in our analyses as shown in the DO database for the example disease—disorders of lipid metabolism perhaps because we only included the strongly correlated 32 pairs from top prevalent 50 disease in the TGA dataset and no associated DO disease were included in our analyses.

Our results also indicated there might be some occurrence order pattern between the strongly associated disease pairs, for example, that the diagnosis of ‘Essential hypertension’ occurred an average of 697 days earlier or at the same time of a diagnosis of ‘Menstrual disorders’ 88% of the time. As shown in Fig. 4, most of the disease pairs have a time delta of more than a year. It is interesting and important to investigate the potential reasons or medical practice considerations. In future work, we will investigate the temporal relationships among some diseases by collaborating with clinical faculty who are expert in the specific disease domains. One key strength of this study was the large nationally representative longitudinal dataset.

For the disease comorbidities analysis, it is important and interesting to further investigate the potential reasons for clustering. For example, heart issues and dermatological issues are seen as clusters in real world data experiments. It is because dermatological diseases are often treated with corticosteroid, which are detrimental to the heart muscle. Therefore, disease ontology (DO) might not capture this cluster but the real-world medical data analysis might capture associations mediated by medication usage or other interventional factors. Therefore, the results may be limited without carefully interpreting the disease comorbidities by considering the potential interventional factors.

Time delay distribution of some pairs in Fig. 5 had different patterns. For example, in the pair of (disorders of lipid metabolism, diabetes mellitus without complication), the blue curve is relatively flat which indicated that there was less likely (no peeks) to have a certain time delay within 3 years for the case of Disorders of lipid metabolism occurred first and then Diabetes mellitus without complication occurred second. On the other hand, the orange curve showed that there was more likely to have a certain time delay (peek) for the co-occurrence of diabetes mellitus without complication first and disorders of lipid metabolism later.

## Limitations

There are limitations to this study. First, in order to study the associations between different diseases, patients with less than five different CCS codes was not included in our analyses. Our conclusions and findings were based on patients with more than five different CCS diagnosis codes. Second, the data registry used represents outpatient clinics participating in one registry. Broadening this registry across more and diverse clinics would obviously produce different associations and results. Third, the temporal relationships among the given disease pairs might be prone to heavy biases due to the following reasons. It is not clear if the diseases of given patients are incident or recurrent. If it is incident, the time delay argument may be more reliable than recurrent diagnoses because the true delay between diseases cannot be well estimated by using the repeated diagnosis. Also, at the point of time of diagnoses, chronic diagnoses could go undiagnosed for a long time. Whereas, acute events are diagnosed more immediately than chronic. Furthermore, hospital codes were not included in this study. Thus, there should be a delay for all codes being recorded; and the delay for chronic conditions can be even greater. The reason is that when faced with acute conditions, the patient might go to the emergency department as opposed to the clinic. Therefore, it may be more reasonable to investigate the chronic conditions and immediate infections/acute conditions separately.

## Conclusions

Understanding these relationships could potentially increase awareness among healthcare providers of co-occurring conditions and facilitate earlier diagnosis and treatment of patients.

It is crucial to investigate disease associations and better understand those co-occurrences may help providers to decrease the incidence of commodities by taking extra steps for control and management. Thus, it might potentially improve patient health and decrease the clinical burden and cost on the health systems caused by other associated diseases.

## Supplementary Information

Supplementary Information.
