# Supplementary material for: Discovering disease–disease associations using electronic health records in The Guideline Advantage (TGA) dataset
Source: Sci Rep. 2021 Oct 25;11:20969. doi: 10.1038/s41598-021-00345-z (PMC8547216; doi:10.1038/s41598-021-00345-z)
Supplement: Supplementary file 1 — Supplementary Information. [file 41598_2021_345_MOESM1_ESM.docx]

**Supplementary Materials**

**Table S1. CCS disease group statistics for all the studied 50 CCS Codes.**

| CCS Code | CCS Code Description | n (%) |
| --- | --- | --- |
| 98 | Essential hypertension | 71895 (43.4) |
| 53 | Disorders of lipid metabolism | 65622 (39.6) |
| 10 | Immunizations and screening for infectious disease | 59050 (35.6) |
| 58 | Other nutritional; endocrine; and metabolic disorders | 55365 (33.4) |
| 258 | Other screening for suspected conditions (not mental disorders or infectious disease) | 52225 (31.5) |
| 126 | Other upper respiratory infections | 47656 (28.8) |
| 134 | Other upper respiratory disease | 38246 (23.1) |
| 133 | Other lower respiratory disease | 37037 (22.3) |
| 205 | Spondylosis; intervertebral disc disorders; other back problems | 36374 (21.9) |
| 663 | Screening and history of mental health and substance abuse codes | 36373 (21.9) |
| 211 | Other connective tissue disease | 34923 (21.1) |
| 49 | Diabetes mellitus without complication | 34293 (20.7) |
| 204 | Other non-traumatic joint disorders | 33116 (20.0) |
| 200 | Other skin disorders | 32186 (19.4) |
| 657 | Mood disorders | 31797 (19.2) |
| 138 | Esophageal disorders | 28076 (16.9) |
| 117 | Other circulatory disease | 27552 (16.6) |
| 106 | Cardiac dysrhythmias | 27397 (16.5) |
| 163 | Genitourinary symptoms and ill-defined conditions | 25971 (15.7) |
| 253 | Allergic reactions | 25256 (15.2) |
| 155 | Other gastrointestinal disorders | 23390 (14.1) |
| 651 | Anxiety disorders | 22712 (13.7) |
| 59 | Deficiency and other anemia | 21717 (13.1) |
| 4 | Mycoses | 21634 (13.1) |
| 101 | Coronary atherosclerosis and other heart disease | 21261 (12.8) |
| 102 | Nonspecific chest pain | 20260 (12.2) |
| 95 | Other nervous system disorders | 20113 (12.1) |
| 128 | Asthma | 19931 (12.0) |
| 251 | Abdominal pain | 19834 (12.0) |
| 257 | Other aftercare | 18574 (11.2) |
| 252 | Malaise and fatigue | 18520 (11.2) |
| 48 | Thyroid disorders | 16552 (10.0) |
| 94 | Other ear and sense organ disorders | 16372 (9.9) |
| 84 | Headache; including migraine | 16148 (9.7) |
| 92 | Otitis media and related conditions | 15970 (9.6) |
| 136 | Disorders of teeth and jaw | 15201 (9.2) |
| 127 | Chronic obstructive pulmonary disease and bronchiectasis | 14245 (8.6) |
| 197 | Skin and subcutaneous tissue infections | 13852 (8.4) |
| 7 | Viral infection | 13214 (8.0) |
| 159 | Urinary tract infections | 12594 (7.6) |
| 171 | Menstrual disorders | 12478 (7.5) |
| 52 | Nutritional deficiencies | 12024 (7.3) |
| 176 | Contraceptive and procreative management | 11460 (6.9) |
| 175 | Other female genital disorders | 11280 (6.8) |
| 203 | Osteoarthritis | 10962 (6.6) |
| 93 | Conditions associated with dizziness or vertigo | 10956 (6.6) |
| 96 | Heart valve disorders | 10910 (6.6) |
| 50 | Diabetes mellitus with complications | 10556 (6.4) |
| 108 | Congestive heart failure; nonhypertensive | 10128 (6.1) |
| 89 | Blindness and vision defects | 9839 (5.9) |

*
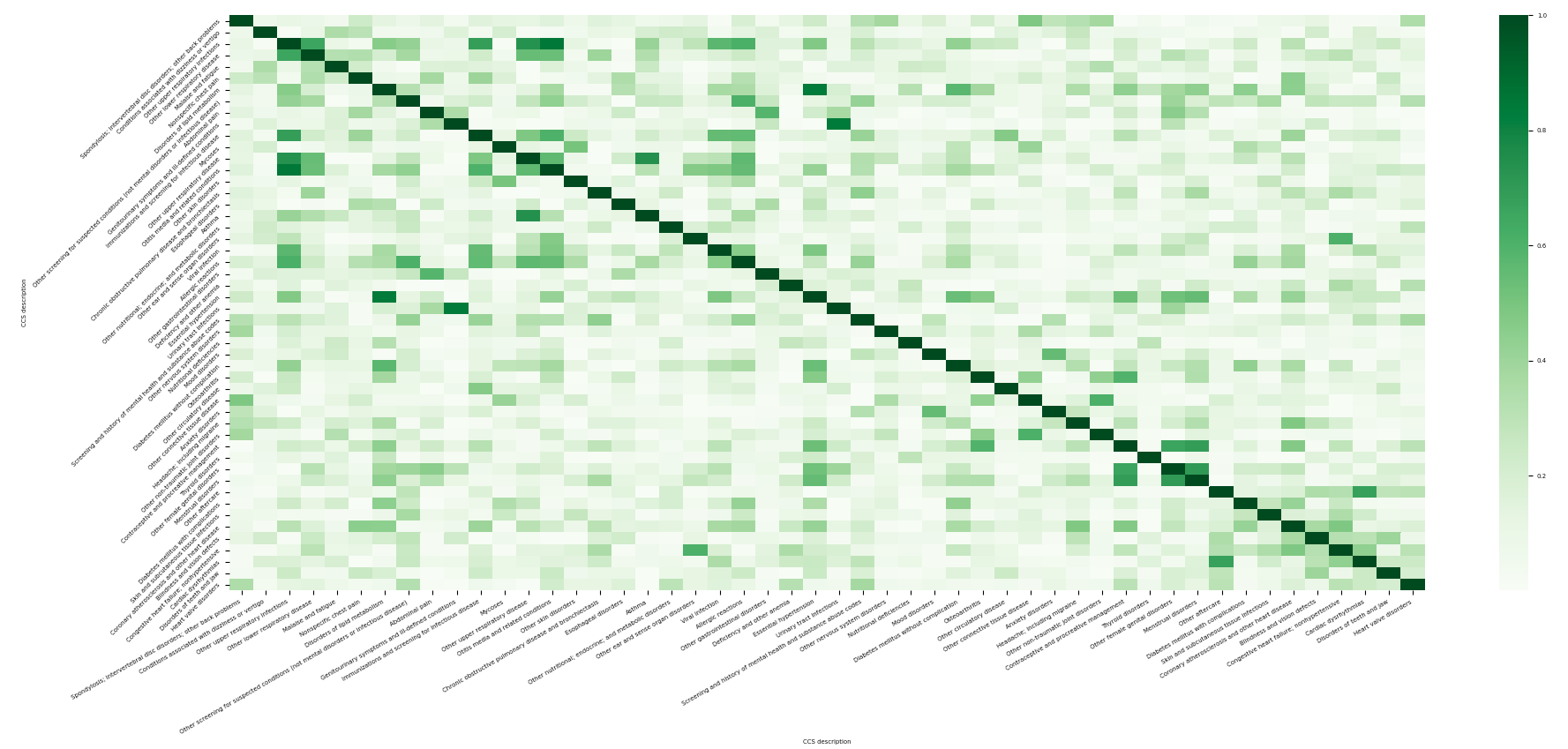
*
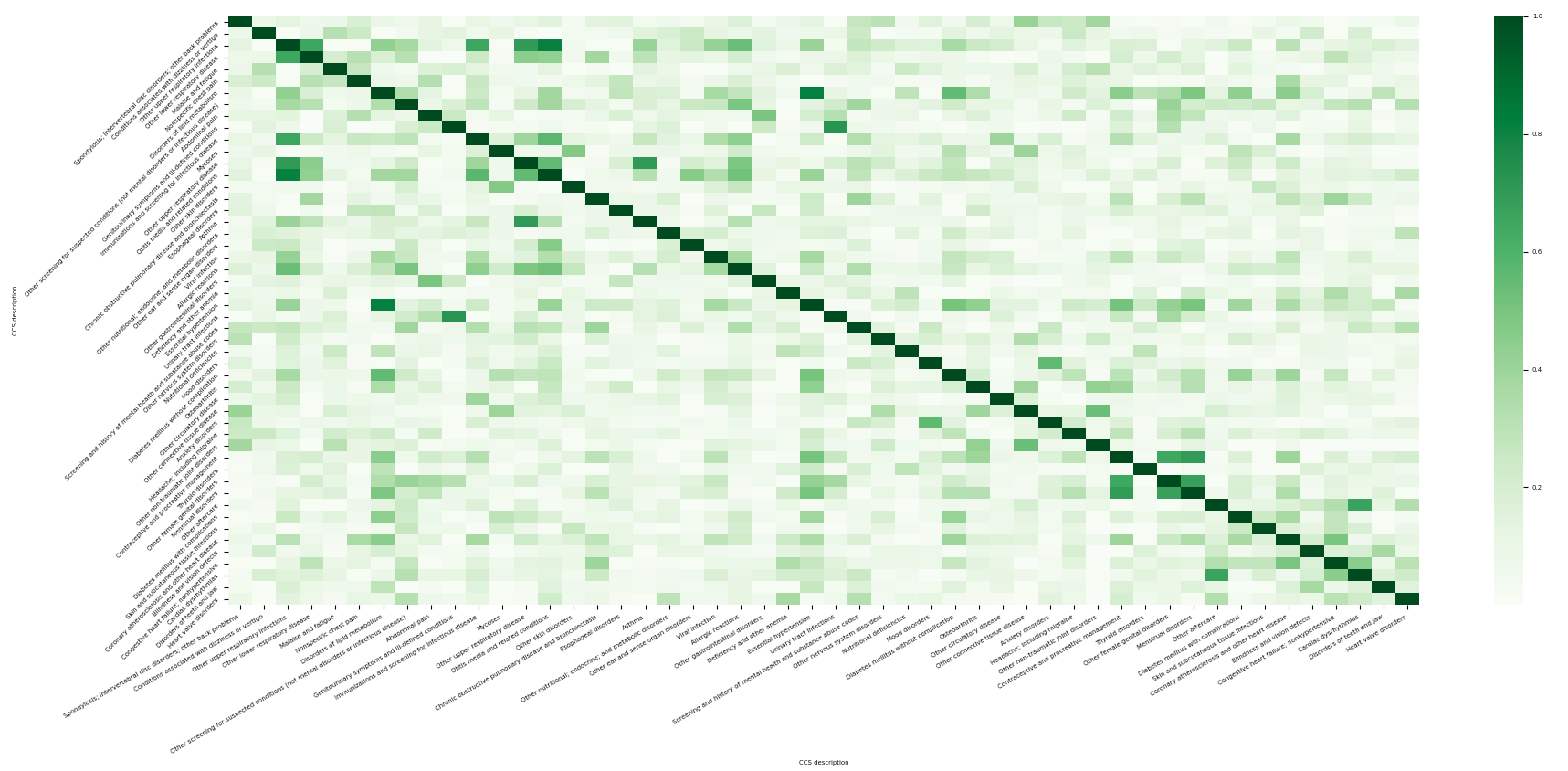


a b


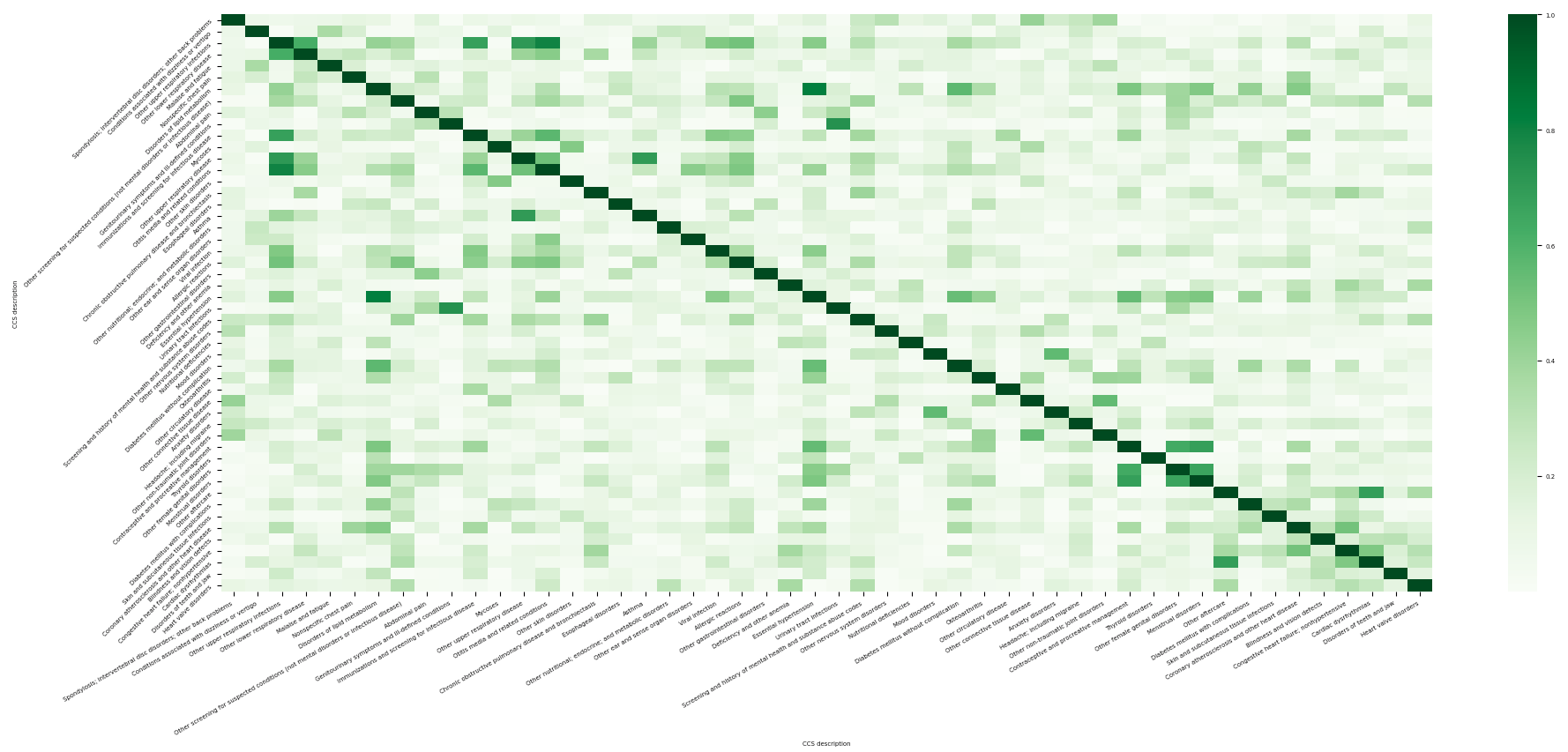

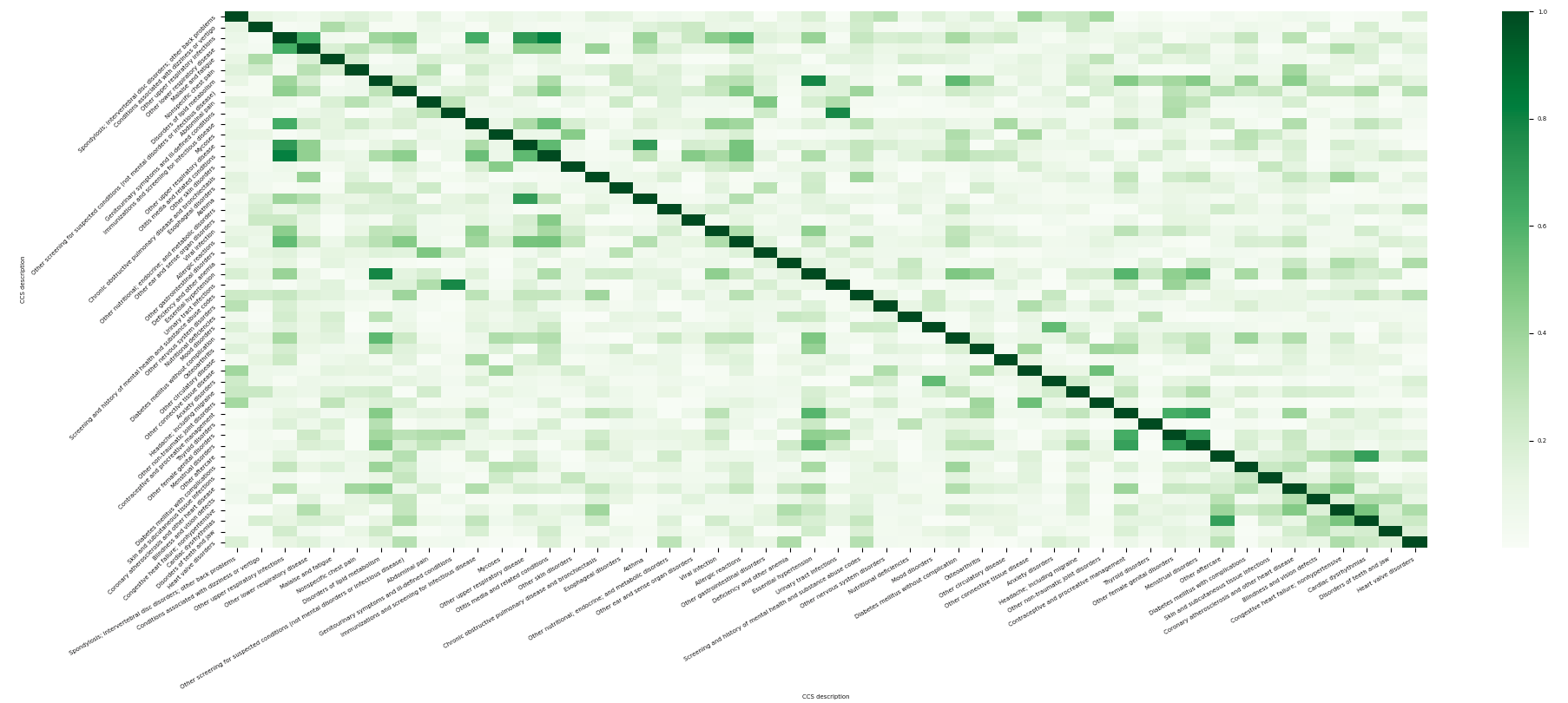


c d


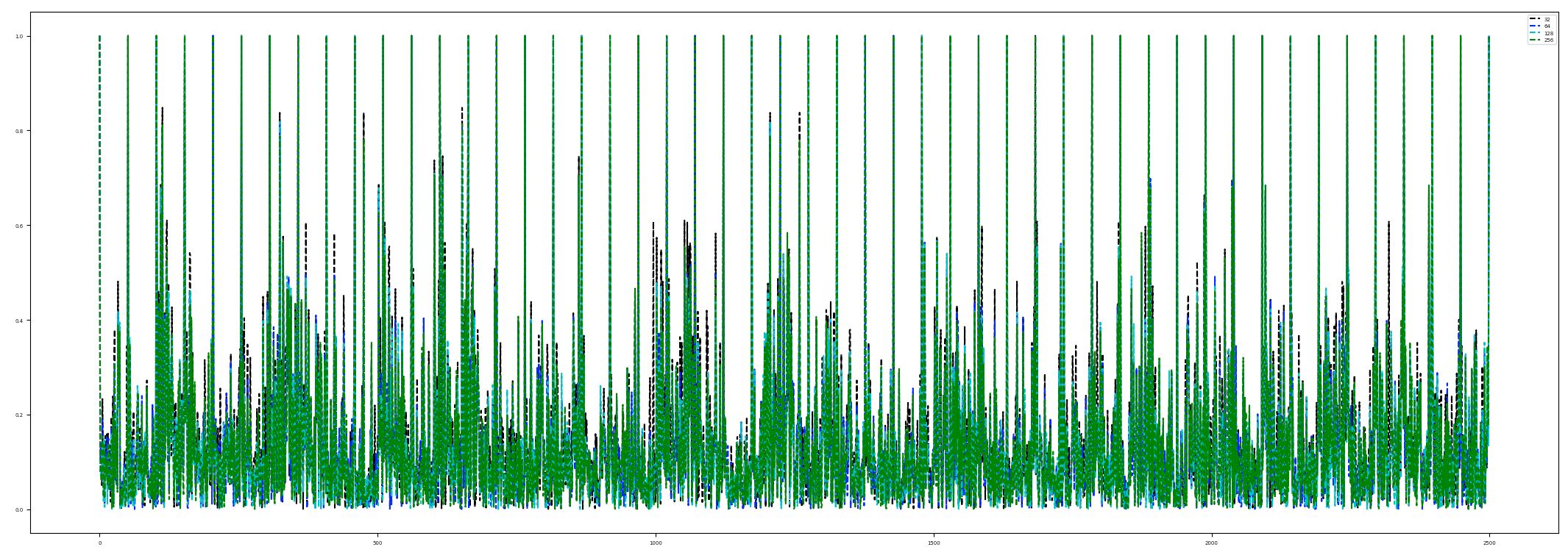


e

**Figure S1.** Correlation analysis of the 50 CCS diagnosis codes for 4 embedding sizes of 32 (a), 64 (b), 128 (c), and 256 (d). Last row figure (e) is for the correlation coefficient matrix by line plots for the 4 cases. Figures were generated by using Python 3.6.8 with package of Matplotlib.
